# You have the microbiome you deserve

**DOI:** 10.1017/gmb.2020.3

**Published:** 2020-08-27

**Authors:** Colin Hill

**Affiliations:** APC Microbiome Ireland and School of Microbiology, University College Cork, Cork, Ireland

**Keywords:** virome, bacteriome, diversity, therapeutics

## Abstract

The human microbiome is one of the most exciting areas of microbiology. From a starting point of tens of papers annually a couple of decades ago, there are now thousands of papers published every year on the microbiome. Huge strides have been made in terms of defining the individual members of complex human microbiomes from different body sites. The individuality and diversity of the human microbiome almost surpasses our ability to comprehend it. Advances in metagenomics and computational sciences have increased the complexity of the field, while at the same time we have moved from regarding the human microbiome as a benign passenger to a situation where it has been linked to almost every chronic disease, including obesity, cancer and infectious disease. The microbiome tantalizes us with the promise of novel therapeutic molecules and modalities for a range of intractable diseases. And yet, very few microbiome-based therapies have made it to the clinic or the pharmacy and we still cannot really define a healthy microbiome. We are entering the most exciting phase of microbiome research, as we develop effective, evidence-based interventions to preserve and restore human health. But we need rigour and numeracy if we are to realize this vision.

Consider your microbiome. Not *the* microbiome in the abstract, but *your* microbiome. How did you come to assemble the enormously diverse community of microbes that live in and on your body? Have you got the ‘right’ ones in the right balance? Is your microbiome helping to maintain your health, is it contributing to ongoing episodes of ill-health, or will it someday lead to your premature death? In 1934 the Dutch scientists Baas-Becking (1934) and deWit and Bouvier (2006) wrote ‘*Everything is everywhere, but, the environment selects*’. This simple statement should resonate with every scientist contemplating the human microbiome. Like many profound and simple statements, it captures the essence of a concept, but it is not absolutely true. In your life, to this point, you have not encountered ‘everything’ in the microbial world and so you have only been able to ‘select’ from those microbes that you have contacted. It is estimated that there are a trillion microbial species on earth, of which only about 10,000 have been cultured (Locey and Lennon, 2016). You will probably have encountered many thousands of these species (possibly millions of strains) and so it is not surprising that your incredibly complex human ecosystem with its many exposed and internal niches has selected an enormously complex community of microorganisms from ‘everything’ you have encountered since your conception. Many ecological dramas will have been played out in and on your body. There will have been founder effects, stochastic encounters, invasions and infiltrations, *force majeure* events (antibiotics, surgery and cancer), while many microbial and microbe–host alliances will have been formed and disrupted – a veritable microbial Game of Thrones.

At this moment in time, you have assembled ‘your’ microbiome, and as stated in the title to this piece, it is the microbiome you ‘deserve’ based on your life to date. Your unique combination of genetics, birth mode, family members, exposure to animals, environment, diet, medical history and lifestyle has selected your microbiome and it is now in a particular conformation, both compositionally and functionally (Figure 1). This complexity makes it difficult for us to understand the roles of the individual microbes and communities that make up our many different sub-microbiomes distributed across our various body sites. We cannot always discern the function of each microbial member, and whether microbe X in one individual plays a role that is performed by microbe Y in another. Frustratingly, we microbiome scientists cannot really tell you whether you have a ‘good’ or a ‘bad’ microbiome. We can measure aspects like diversity and richness, we can ‘name’ the inhabitants and count their genes and place them into functional categories. We can build extraordinarily complex figures (PCoA’s, heatmaps and networks) and compare your microbiome to those of other individuals, but with very rare exceptions we cannot really tell if you would benefit from a specific change, nor can we precisely sculpt your microbiome in a predictable fashion. Some of my microbiome colleagues might interject at this point that we do have some general principles, we know that diversity is a good thing (except in those body cavities where it is not), and community stability and resilience is a good thing (unless you have a disease in which your microbiome may be driving the pathology). But these are hypotheses, and almost every control group seems to have those frustrating individuals with a microbiome that seems to fit better with the disease group, and *vice-versa.* We have become used to seeing scatter plots of diversity or richness in disease versus control where the most striking aspect to the casual viewer is the overlap between the groups, despite the statistical significance captured in the low *P* values decorating each figure. We realized very early that the contribution of the microbiome to health and disease is not a paradigm that follows simple Koch’s postulates, no matter how hard we strive to understand it in this fashion.

**Figure 1:**
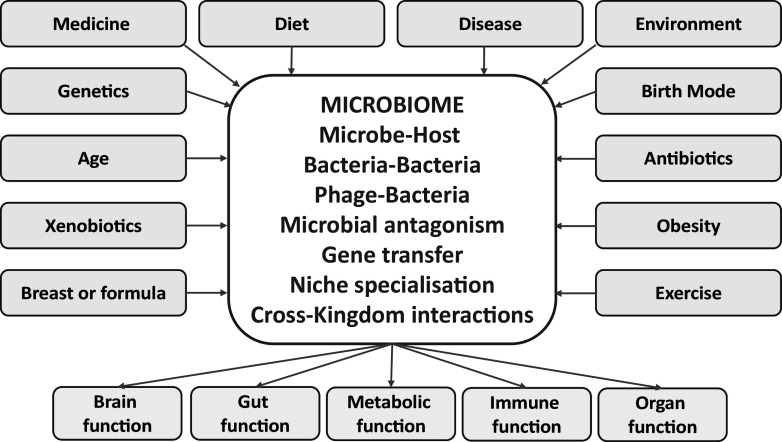
Many factors shape the microbiome, both external and internal. The resulting final composition and functionality will affect health outcomes across many organs.

Together with my colleague Fergus Shanahan, I have already railed against the lack of precise language, numeracy and literacy in microbiome science (Shanahan & Hill, 2019), and so I will not rehash that argument here in detail. I do want to once again call out the term ‘dysbiosis’ in the way in which it is currently used by many scientists. It may well be that the ‘different’ or ‘altered’ microbiome in someone with a specific disease or syndrome is neither causal nor contributory (the connotation conveyed by the word dysbiosis). One could equally argue that an altered microbiome may even be protective against disease progression in some or many instances. What is certainly true is that the altered microbiome has been selected by the changed conditions in the diseased gut and we should only seek to change it if it we can be sure that such a change would be beneficial. *First do no harm* should be as applicable in microbiome-based therapies as in any other aspect of medicine. It may well be that by trying to manipulate an individual microbiome we are working against ecological forces in trying to alter what Nature has already selected. This is almost certainly why most probiotics or live biotherapeutics are unable to colonize consumers or patients. On the other hand, we can certainly view the delivery of live microbes (either as single strains or consortia) as providing alternatives that the ecosystem can ‘choose’ to select or ignore. In any event, some of the benefits of these microbial therapies probably result from interactions with the enteric immune or nervous systems rather than any direct impact on the microbiome. Equally, when we impose an intervention (e.g. antibiotics) directed at the gut microbiome and it inevitably changes in response, this should not really be characterized as ‘dysbiosis’. We have changed the environment, which has then selected for a different microbial community structure – there is nothing dysbiotic about that.

Numeracy is another problem for the microbiome field. We rely too much on relative abundances, making us one of very few scientific disciplines that does not demand absolute numbers as the bedrock of all further analytical steps. Sampling is yet another problem. How representative is a faecal sample of the human gut microbiome? It is trivializing the field when some scientists (*mea culpa*) refer to the relative abundance of 16S rRNA amplicons in a faecal sample as the ‘gut microbiome’. We can no longer ignore the virome, the mycome, the archaeome and the protozome. We must take on the challenge of looking at multiple body sites and looking longitudinally and radially through the gut and other body sites. We must include standards or use protocols that give us absolute numbers. How can we compare the microbial communities in two individuals in any meaningful fashion if we are not aware that one of the subjects has ten times more microbes than the other?

I have presented a somewhat gloomy view of microbiome science and scientists, but that is not my intention. I believe in the potential of the microbiome both as a therapeutic target and a rich repository of novel therapies. I believe that the microbiome plays a central role in our health and that this amazing superorganism we call a human is a combination of the macroscopic and microscopic. The evidence for this belief is borne out by too many examples to list here, but one particularly convincing line of evidence is when faecal transfer to naïve animals is followed by the development of symptoms consistent with the disease of the donor. But this belief is not inconsistent with the view that microbiome research must be a literate and numerate science, driven by testable hypotheses and rigorous analysis. Many scientists are doing exactly that, but it does no favours to the field when the microbiome is linked by the flimsiest evidence (an altered microbial community) to every disease, every syndrome and to every aspect of life. As we move through life things change in our bodies, our diet and our surroundings, and so the microbiome, which is ruthlessly and dispassionately selected by our environment, also changes. This is to be expected, not trumpeted as proof of yet another microbiome-associated disease. It is no wonder that nonmicrobiome scientists roll their eyes at every new breakthrough. There is no shortcut for microbiome science on the long and rigorous path to improve human health *via* the clinic or the food chain. I believe that the human microbiome is one of the most exciting areas of biology and offers the prospect of significant breakthroughs in our understanding of human health and wellbeing, but we must ensure that studying the microbiome remains a science and not a belief system.
